# Relationship between genetic diversity and morpho-functional characteristics of flight-related traits in *Triatoma garciabesi* (Hemiptera: Reduviidae)

**DOI:** 10.1186/s13071-024-06211-x

**Published:** 2024-03-18

**Authors:** Thaiane Verly, Sebastián Pita, Ana Laura Carbajal-de-la-Fuente, Gabriela Burgueño-Rodríguez, Romina V. Piccinali, Federico G. Fiad, Néstor Ríos, Francisco Panzera, Patricia Lobbia, Paz Sánchez-Casaccia, Antonieta Rojas de Arias, María José Cavallo, Gisel V. Gigena, Claudia S. Rodríguez, Julieta Nattero

**Affiliations:** 1 Centro Nacional de Diagnóstico e Investigación en Endemo-Epidemias (CeNDIE), Administración Nacional de Laboratorios e Institutos de Salud “Dr. Carlos Malbrán” (ANLIS), Buenos Aires, Argentina; 2 Consejo Nacional de Investigaciones Científicas y Técnicas (CONICET), Buenos Aires, Argentina; 3 Sección Genética Evolutiva, Facultad de Ciencias, Universidad de la República, Montevideo, Uruguay; 4 Departamento de Ecología Genética y Evolución, Laboratorio de Eco-Epidemiología, Facultad de Ciencias Exactas y Naturales, Universidad de Buenos Aires, Buenos Aires, Argentina; 5 Instituto de Ecología, Genética y Evolución (IEGEBA), CONICET/Universidad de Buenos Aires, Buenos Aires, Argentina; 6 Cátedras de Introducción a la Biología y Morfología Animal, Instituto de Investigaciones Biológicas y Tecnológicas (IIByT), Facultad de Ciencias Exactas Físicas y Naturales, Consejo Nacional de Investigaciones Científicas y Técnicas (CONICET)/Universidad Nacional de Córdoba, Córdoba, Argentina; 7 Unidad Operativa de Vectores y Ambiente (UnOVE), Administración Nacional de Laboratorios e Institutos de Salud “Dr. Carlos Malbrán”, Centro Nacional de Diagnostico e Investigación en Endemo-Epidemias (CeNDIE), Córdoba, Argentina; 8 Centro para el Desarrollo de la Investigación Científica (CEDIC), Asunción, Paraguay; 9 Centro Regional de Energía y Ambiente Para el Desarrollo Sustentable (CREAS-CONICET), Universidad Nacional de Catamarca (UNCA), San Fernando del Valle de Catamarca, Catamarca Argentina

**Keywords:** Cytochrome *c* oxidase I gene, Hemelytra, Head, Pronotum, Geometric morphometry, Size and shape variation

## Abstract

**Background:**

*Triatoma garciabesi*, a potential vector of the parasitic protozoan *Trypanosoma cruzi*, which is the causative agent of Chagas disease, is common in peridomestic and wild environments and found throughout northwestern and central Argentina, western Paraguay and the Bolivian Chaco. Genetic differentiation of a species across its range can help to understand dispersal patterns and connectivity between habitats. Dispersal by flight is considered to be the main active dispersal strategy used by triatomines. In particular, the morphological structure of the hemelytra is associated with their function. The aim of this study was to understand how genetic diversity is structured, how morphological variation of dispersal-related traits varies with genetic diversity and how the morphological characteristics of dispersal-related traits may explain the current distribution of genetic lineages in this species.

**Methods:**

Males from 24 populations of *T. garciabesi* across its distribution range were examined. The cytochrome* c* oxidase I gene (*coI*) was used for genetic diversity analyses. A geometric morphometric method based on landmarks was used for morpho-functional analysis of the hemelytra. Centroid size (CS) and shape of the forewing, and contour of both parts of the forewing, the head and the pronotum were characterised. Length and area of the forewing were measured to estimate the aspect ratio.

**Results:**

The morphometric and phylogenetic analysis identified two distinct lineages, namely the Eastern and Western lineages, which coincide with different ecological regions. The Eastern lineage is found exclusively in the eastern region of Argentina (Chaco and Formosa provinces), whereas the Western lineage is prevalent in the rest of the geographical range of the species. CS, shape and aspect ratio of the hemelytra differed between lineages. The stiff portion of the forewing was more developed in the Eastern lineage. The shape of both portions of the hemelytra were significantly different between lineages, and the shape of the head and pronotum differed between lineages.

**Conclusions:**

The results provide preliminary insights into the evolution and diversification of *T. garciabesi*. Variation in the forewing, pronotum and head is congruent with genetic divergence. Consistent with genetic divergence, morphometry variation was clustered according to lineages, with congruent variation in the size and shape of the forewing, pronotum and head.

**Graphical Abstract:**

**Figure:**
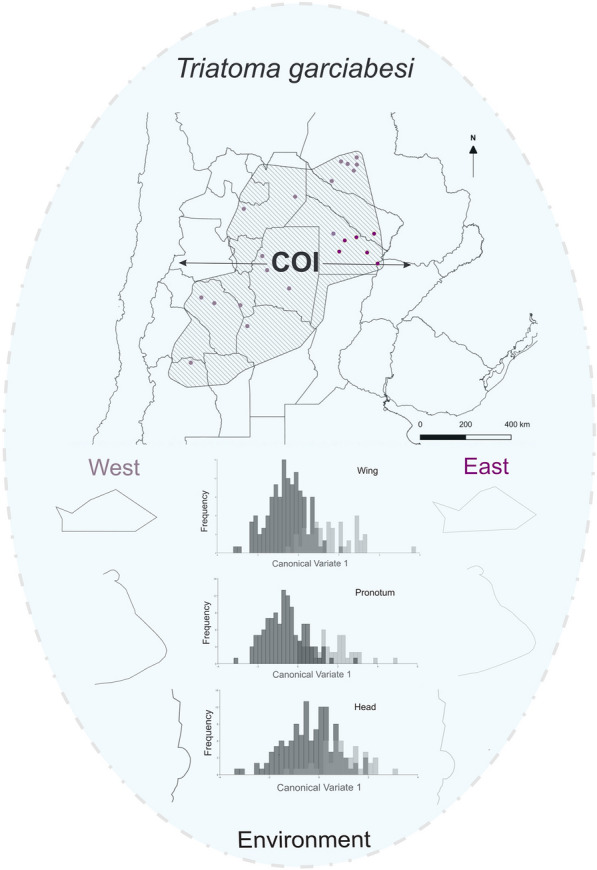

**Supplementary Information:**

The online version contains supplementary material available at 10.1186/s13071-024-06211-x.

## Background

Chagas disease is an anthropozoonosis caused by the protozoan *Trypanosoma cruzi* Chagas 1909 (Kinetoplastea: Trypanosomatida) [1] that affects 6–7 million people worldwide, mainly concentrated in the 21 Latin American countries and territories [2]. Triatomines, the vectors of *T. cruzi*, are hemipteran bugs belonging to the subfamily Triatominae (Hemiptera: Heteroptera: Reduviidae) [3]. The genus *Triatoma* includes 84 species grouped in eight complexes and 14 subcomplexes and is the most diverse and one of the most important genus from an epidemiological point of view [4]. Within the *Triatoma* genus, the Sordida subcomplex is composed of *Triatoma garciabesi* Carcavallo, Cichero, Martínez, Prosen, Ronderos, 1967, *Triatoma sordida* Stål, 1859 sensu lato (*T. sordida* Stål, 1859 s.l.) and* Triatoma rosai* Alevi, Oliveira, Garcia, Cristal, Delgado, Bittinelli, Reis, Ravazi, Oliveira, Galvăo, Azeredo-Oliveira, Madeira, 2020, among other species [5, 6]. The original grouping of these species was based on shared morphological characters, which were also used to include other species such as *Triatoma guasayana* Wygodzinsky & Abalos, 1949 [5]. Nevertheless, genetic data, including chromosomal and DNA sequences [4, 6, 7], wing morphometry and molecular analyses [8] and cuticle hydrocarbon analysis [9] have contributed to the current configuration of the subcomplex, in which *T. guasayana* is not included.

*Triatoma garciabesi* has historically been considered to be a single species or synonymised with *T. sordida* due to morphological similarities and partial overlap in geographical distribution. Its validation as a separate species was published in 1967 and was based on the observation of morphological differences in the head and phallic structures [10]. However, *T. garciabesi* was synonymised with *T. sordida* by Lent and Wygodzinky in 1979 [3], with these authors concluding that the phenotypic differences were not sufficient to consider two distinct species. Regardless, two decades later, evidence based on microhabitat characters, male genitalia and cytogenetics reaffirmed its taxonomic status as *T. garciabesi* [for review see 11]. Various approaches have been used to justify the taxonomic validity of this species, including geographical distribution [7, 10, 12], morphology of the head, eggs and genitalia [13, 14], morphometry of the forewings, head and pronotum [8, 14, 15], cuticular hydrocarbons [9], isozymes [17], cytogenetic characteristics [7] and molecular analyses [7, 8, 18]. *Triatoma garciabesi* is usually found in peridomestic environments, mainly associated with trees where chickens roost [19]. The species occasionally invades rural houses [20, 21] and has been recorded in wild environments [22, 23]. Geographical distribution records based on morphological characteristics indicate that this species is present in the semi-arid regions of northwestern and central Argentina and western Paraguay [7, 15, 16, 24]. Based on cytogenetic analysis, its distribution has been extended to the Bolivian Chaco (Santa Cruz department), but there are no records of its presence in other areas of Bolivia [7]. According to an ecological niche study, the drier areas of northwestern Argentina provide ideal conditions for the occurrence of *T. garciabesi* [15].

The analysis of nuclear and mitochondrial DNA (mtDNA) sequences has proven to be suitable for determining genetic diversity and population structure of triatomines [25–27]. In other insects, the mitochondrial *cytochrome c* *oxidase* *I* gene (*coI*) has been widely used to improved understanding of species diversity and delimitation [28]. Assessing the genetic differentiation of a species across its geographical range can provide insights into dispersal patterns and habitat connectivity. Dispersal also plays a significant role in shaping the spatiotemporal distribution of a species' genetic diversity [29]. Active dispersal tendencies of a species or its populations evolve according to changes in locomotory and navigation traits [30]. The relationship between the shape and function of insect forewings would be subject to strong selective pressures, with not only behavioural activities like oviposition and foraging preferences being taken into consideration, but also flight-related characteristics that enhance mobility within a specific environmental context [30, 31]. In triatomines, flight is considered to be the most important active dispersal mechanism [32, 33]. For *Triatoma infestans* Klug, 1834, flight distances of between 200 m [34] and 1500 m [35] have been reported. Hemipteran forewings, named hemelytra, are composed of a stiff proximal region and a more flexible membranous apex. The morphological structure of the hemelytra is linked to their function: the membranous part can be deformed by aerodynamic and inertial forces, and the stiff part limits deformations and provides support [36]. The shape, size and development of the parts of the insect forewing define the characteristics related to flight efficiency. For example, in butterflies and damsflies, elongated and narrow wings determine greater deformation capacity with lower energy cost for long distance flights, whereas short and broad forewings are compatible with short, slow and less stable flight [37, 38]. Although wing morphology in triatomines is quite different, for *T. guasayana*, elongated and thin wings were suggested to allow sustained long-duration flights, and short and wide wings would be related to short flights [39]. Wing aspect (4 × [wing length^2^/wing area]) is also an indicator of flight capacity, with individuals having high values of this parameter being able to fly faster and farther than individuals with low wing aspect values [40].

In insects, the ability to orientate in space and in relation to the specific environmental cues (seasonal events, daily cycles and specific temporal cues) contributes to the navigational abilities, which are linked to sensory and spatial cognitive abilities. All of these abilities are provided by organs, such as eyes for vision, antennae for olfaction and the brain for celestial navigation and cognitive maps (e.g. [30]). In several insects, changes in head phenotype have been associated with flight capacity [30, 41, 42]. In the triatomines *Mepraia spinolai* Porter, 1934 and *T. guasayana*, some characteristics, such as narrow head with highly developed compound eyes, are considered to be associated with flight dispersal [41–43]. When developed, flight muscles are located under the pronotum. In some triatomines, an increased dispersal capacity was found to coincide with a wide thorax, despite the intermediate size of forewings. A wide thorax may imply highly developed forewing muscles [44].

Flight dispersal is a complex and plastic attribute determined by a suite of morphological, physiological and behavioural characteristics [29], all of which have genetic basis and are influenced by the environment [45]. Similar to several other insects, triatomines respond rapidly to environmental changes; therefore, they are considered a suitable model for studying the effects of genetic diversity on changes associated with dispersal. For example, evolutionary changes in flight morphology have been linked to range expansion, colonisation success, habitat fragmentation and migration. The aim of the present study was to understand the structuring of genetic diversity of *T. garciabesi*, the variation in morphology of dispersal-related traits in relation to genetic diversity and the relationship between morphological characteristics of dispersal-related traits and the current distribution of genetic lineages in this species.

## Methods

### Insects and collection sites

**Fig. 1:**
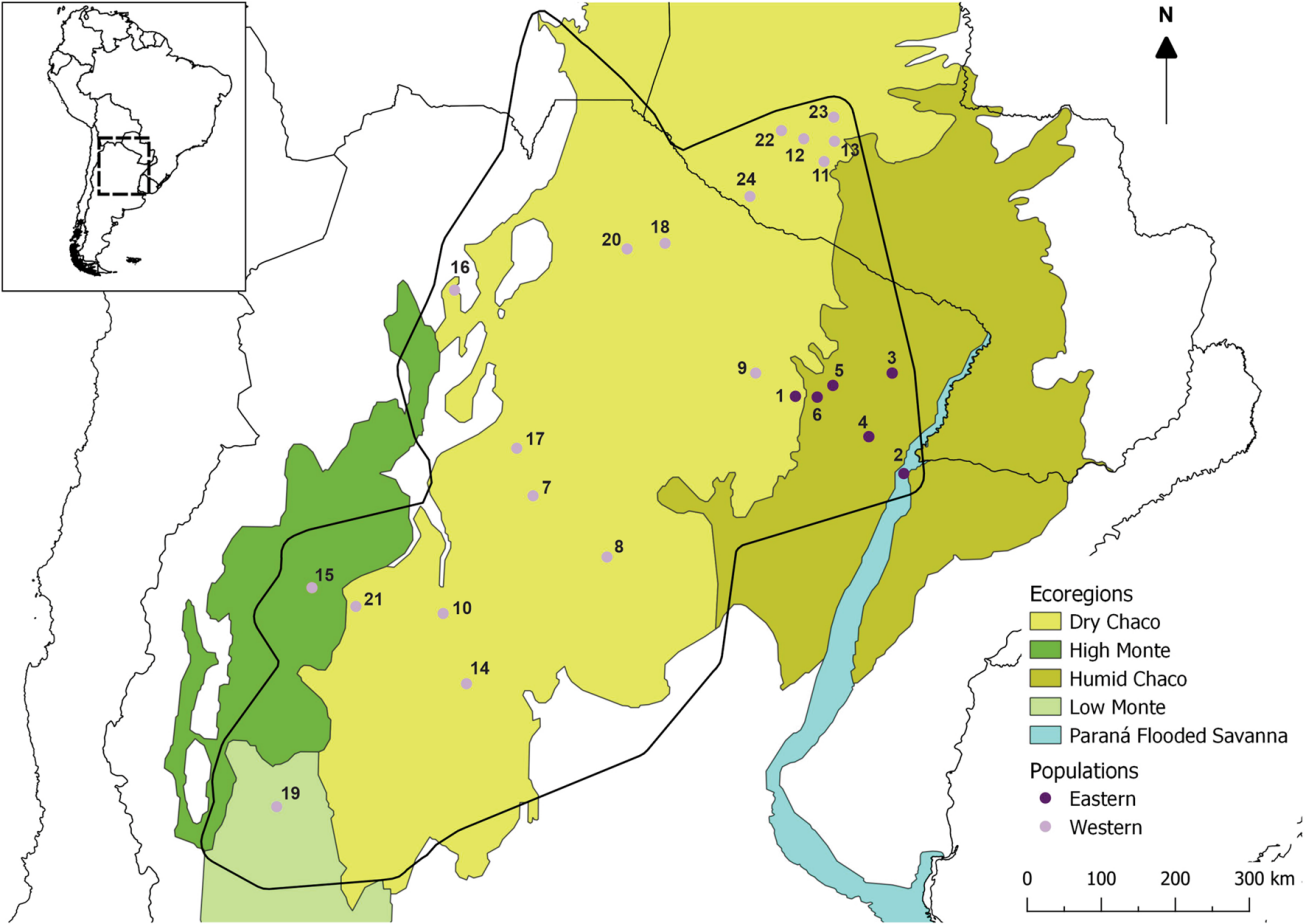
Distribution area of *Triatoma garciabesi* based on Ceccarelli et al. [24] and extended to the Paraguayan populations included in this study. Filled circles indicate the location of the studied populations, with dark-purple circles corresponding to the populations assigned to the Eastern lineage by *coI* analysis and lilac circles corresponding to populations assigned to the Western lineage by *coI* analysis. Numbers correspond to the population code in Table 1. Ecoregions are according to Dinerstein et al. [88] (https://ecoregions.appspot.com/).* coI*, Cytochrome* c* oxidase I gene

*Triatoma garciabesi* male bugs were collected from 24 populations found in Argentina and Paraguay (Table 1; Fig. 1). We included only males in the study because their sample size was larger than that of females and because sexual dimorphism has been observed in some populations from Argentina (Fiad et al. unpublished data). The collected specimens were first identified as males based on morphological features and then the results were confirmed by molecular analyses for one or two individuals from each population (see section DNA sequence analysis). The localities included in the study cover almost all *T. garciabesi*’s distribution area (Fig. 1). All populations were collected from the field, except for one colony, Balbuena, which consisted of a population reared for one generation in the laboratory (Table 1). Individuals from Balbuena colony were supplied by the “Unidad Operativa de Vectores y Ambiente (UnOVE)”, “Centro Nacional de Diagnóstico e Investigación en Endemo-Epidemias (CeNDIE)”, which is part of the “Administración Nacional de Laboratorios e Institutos de Salud ‘Dr. Carlos Malbrán’ (ANLIS), from Santa María de Punilla, Córdoba, Argentina. In the field, *T. garciabesi* populations were collected from peridomestic structures, except for those from “Reserva Natural Bosques Telteca”, Mendoza province, Argentina, which were collected from sylvatic habitats. The distribution of *T. garciabesi* encompasses five ecoregions (Fig. 1): Humid Chaco, Dry Chaco, High Monte, Low Monte and Paraná Flooded Savanna (the latter region contains 1 population). Ecoregions are considered to be terrestrial units that contain a geographically distinct set of species, natural communities and environmental conditions [46]. The Humid Chaco ecoregion is an extremely flat plain, with very gentle slopes oriented from west to east. The climate is humid temperate, and rainfall follows a marked longitudinal gradient, with maximum records (exceeding 1300 mm rainfall) in the east [47]. The Dry Chaco ecoregion is characterised by a warm subtropical continental climate, with annual rainfall concentrated in the summer and ranging between 500 and 700 mm [48]. The climate in the High Monte ecoregion is temperate-arid, with annual rainfall of up to 200 mm, while the Low Monte ecoregion is also a temperate-arid climate, with annual rainfall of about 100 mm, occasionally reaching up to 200 mm [48].

**Table 1:** Genetic lineages of *Triatoma garciabesi* populations defined using the *cytochrome c* *oxidase* *I* gene

Population code	Population name	*coI* group	Province/State	Country	Latitude	Longitude	Number of individuals used in the genetic analysis	Number of individuals used in the morphometric analysis
1	3 Isletas	East	Chaco	Argentina	− 26.33	− 60.41	1	8
2	Corrientes	East	Corrientes	Argentina	− 27.46	− 58.83	1	5
3	C. Gral Belgrano	East	Formosa	Argentina	− 26.00	− 59.00	4	21
4	La Esperanza	East	Chaco	Argentina	− 26.92	− 59.34	1	8
5	Lote 4	East	Chaco	Argentina	− 26.18	− 59.86	1	5
6	Maipú	East	Chaco	Argentina	− 26.35	− 60.09	2	6
7	Aguirre	West	Stgo del Estero	Argentina	− 27.79	− 64.26	1	9
8	Avellaneda	West	Stgo del Estero	Argentina	− 28.69	− 63.17	1	10
9	Balbuena	West	Chaco	Argentina	− 26.00	− 61.00	1	9
10	Balde de Punta	West	Catamarca	Argentina	− 29.51	− 65.57	1	17
11	Caacupé	West	Boquerón	Paraguay	− 22.90	− 59.99	1	5
12	Canausa	West	Boquerón	Paraguay	− 22.57	− 60.29	1	5
13	Casuarina	West	Boquerón	Paraguay	− 22.60	− 59.84	1	6
14	Cruz del Eje	West	Córdoba	Argentina	− 30.54	− 65.23	1	15
15	Gral San Martín	West	La Rioja	Argentina	− 29.14	− 67.50	1	9
16	Hickman	West	Salta	Argentina	− 24.78	− 65.41	3	5
17	Loreto	West	Stgo del Estero	Argentina	− 27.10	− 64.50	1	5
18	Pozo Yacaré	West	Formosa	Argentina	− 24.10	− 62.32	1	5
19	Reserva Telteca	West	Mendoza	Argentina	− 32.34	− 68.01	1	6
20	Rivadavia	West	Salta	Argentina	− 24.18	− 62.88	3	8
21	R. Vera Peñaloza	West	La Rioja	Argentina	− 29.41	− 66.85	3	10
22	Sandhort	West	Boquerón	Paraguay	− 22.45	− 60.62	1	7
23	Tiberia	West	Boquerón	Paraguay	− 22.25	− 59.85	1	7
24	Yotoisha	West	Boquerón	Paraguay	− 23.41	− 61.08	1	7
–	–	West	Boquerón	Paraguay	–	–	2	–

All samples were collected in the field, except for individuals from the Balbuena population (first laboratory generation)

*coI* *Cytochrome c* *oxidase* *I* gene

### DNA sequence analysis

A fragment of the mitochondrial *coI* of 34 insects collected from the 24 collection sites was analysed. Due to incongruences and misclassifications within the Sordida subcomplex species in the GenBank nucleotide database, a number of specimens from other* Triatoma* species were taken into account: *T. sordida, T. sordida* La Paz and *T. rosai* individuals; these had previously been determined by Panzera et al. [7] using chromosomal markers. In addition, newly available sequences from *T. guasayana* Genbank Acc. Nos. OR650776 to OR650782, also determined by Panzera et al [7], and a sequence from *Triatoma rubrovaria* Blanchard, 1843 Genbank Acc. No. AF021206 were used as outgroups to the Sordida subcomplex. Total DNA was extracted from the legs fixed in 70% ethanol (3 per specimen) using a standard phenol–chloroform technique procedure. A approximately 624-bp *coI* fragment was amplified by PCR using primers coIb (5′-TAA GCG TCT GGG TAG TCT GAR TAK CG-3′) [49] and ACO (5′-CTT GCA GGA GGA GGA GAY CC-3′) [50]. The cycling parameters for the *coI* gene consisted of 5 min at 95 °C, followed by amplification for 30/35 cycles of 30 s at 95 °C, 30 s at 58 °C/47 °C and 1 min at 72 °C and terminated by 10 min at 72 °C, using the Taq^®^ 2× Master Mix Kit (New England Biolabs, Beverly, MA, USA). The PCR products were sent to Macrogen Inc. (Seoul, Korea) for DNA purification and subsequent sequencing. Both sequence strands were aligned and manually curated by chromatogram evaluation using Chromas (https://technelysium.com.au/wp/chromas/), and then deposited in the GenBank database (http://www.ncbi.nlm.nih.gov), under accession numbers OR650712 to OR650782. Sequence alignment was performed using MAFFT v7.310 [51]. The R package phangorn [52] was used to estimate a phylogenetic tree using the maximum likelihood (ML) method. The tree was bootstrapped 100 times to assess node support. The phangorn JModeltest function was used to find the best-fitting substitution model under the Bayesian Information Criterion (BIC). The phylogenetic trees were visualised and edited in ggtree [53]. The R core base version 4.3.0 ( R Foundation for Statistical Computing, Vienna, Austria), the vegan (https://CRAN.R-project.org/package=vegan), ape v5.7.1 [54] and pegas v1.2 [55] packages were used to calculate the within and intergroup pairwise Kimura two parameter (K-2p) distances [55]. A table and a heatmap were produced with the distance values. In addition, using the K-2p distance matrix, we conducted a multidimensional scaling (MDS) analysis with the base R package stats. The resulting MDS configuration was plotted in a two-dimensional scatter plot. The* x* and* y* coordinates of each point in the plot represented the positions of the individuals in the reduced space, with the distances between the points reflecting their genetic dissimilarities [56]. To determine if the dataset exhibited distinct groupings or clusters based on genetic distances, a clustering analysis was performed on the MDS plot using* k*-means clustering, with the function* k*-means of the R stats package.

In addition, a minimum spanning network (MSN) haplotype network was constructed using PopART software [57].

### Geometric morphometry

**Fig. 2:**
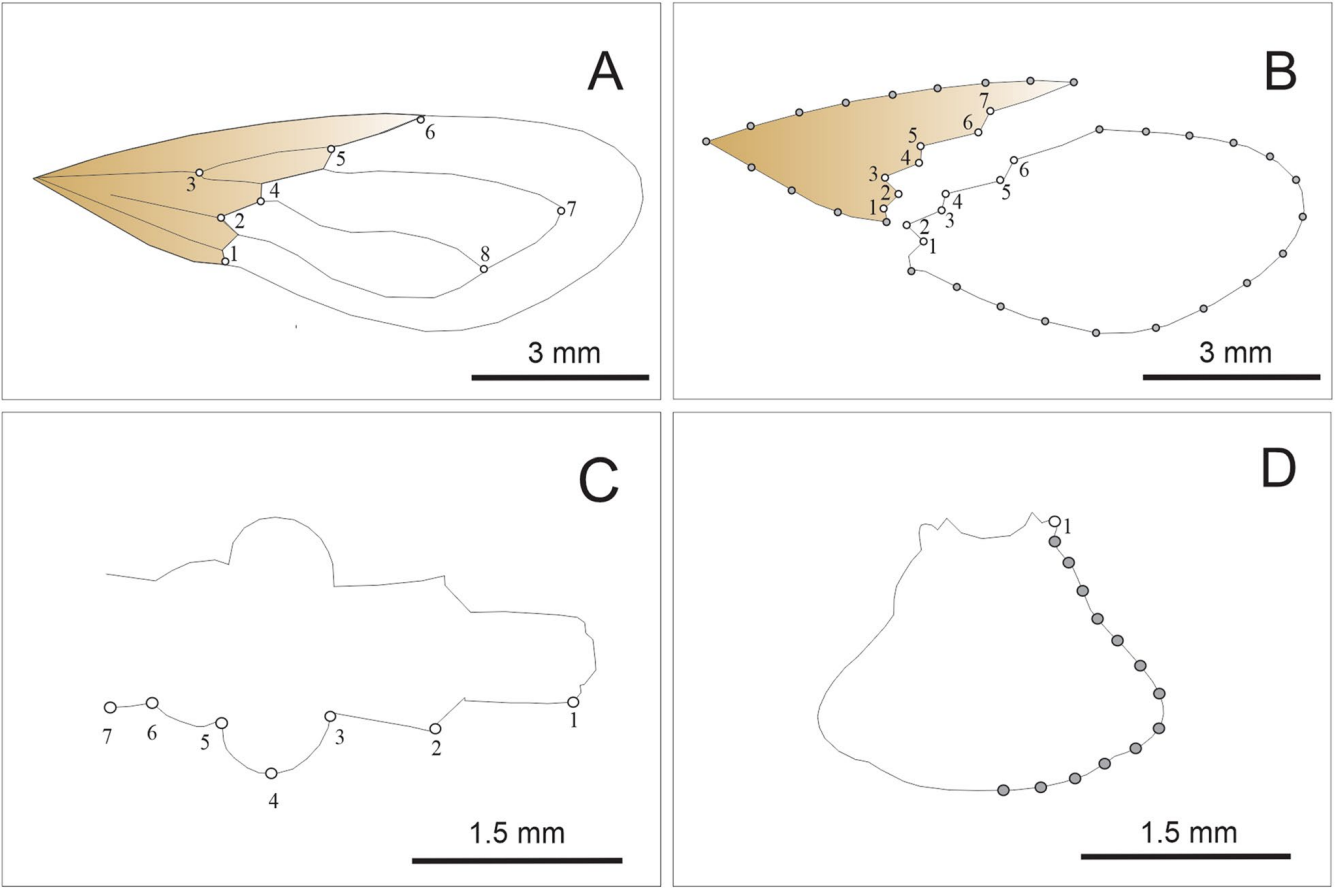
**a** Landmark (open circles) positions for the stiff (brown) and membranous (white) portions of the forewing.** b** Landmark (open circles) and semilandmark (grey-filled circles) positions for the stiff (brown) and membranous (white) portions of the forewing.** c** Landmark positions (open circles) for the head.** d** Landmark (open circles) and semilandmark (grey-filled circles) positions for the pronotum contour, for the studied* Triatoma garciabesi* individuals

Individuals were grouped according to lineages based on their results of the *coI* genetic analyses. A total of 198 individuals belonging to the 24 populations were included in the morphometry analysis (Table 1). For all individuals, digital images of the dorsal view of the right forewing and pronotum and of the ventral view of head were taken using a digital camera (model S9900; Nikon Corp., Tokyo, Japan) mounted on a stereomicroscope (model Stemi SV-11; Carl Zeiss AG, Jena, Germany) at 6× magnification. All images included a reference scale. The maximum forewing length and the total forewing area, which were used to estimate the wing aspect ratio, were measured using ImageJ software version 1.52 (https://imagej.nih.gov/ij/). The wing aspect ratio (4 × [wing length^2^/wing area]; [40]) was compared among lineages using one-way analysis of variance (ANOVA). A quantitative shape analysis was also performed using geometric morphometry based on the statistical analysis of landmark coordinates for the forewing, membranous and stiff portion of the forewing, the head and the pronotum. Landmarks are point locations that are biologically homologous across specimens [58]. Semilandmarks were used to analyse the lateral contour of both the stiff and membranous parts of the forewing and the contour of the pronotum. These parts do not exhibit sufficient homologous points to be used to characterise the morphology of the specimens using traditional landmarks; therefore, the contour of these parts using semilandmarks were quantified [59]. To analyse forewing variation, we included eight type I landmarks of the right forewing (Fig. 2a). Landmarks 1, 2, 4, 5 and 6 are positions between the stiff and membranous portions of the hemelytra. The stiff and membranous parts of the forewing were analysed separately. A combination of landmarks and semilandmarks were used to characterise the contour of these portions of the forewing. For the stiff portion, seven type I landmarks were used to characterise the shape of the boundary between the stiff and membranous parts, and 13 equidistant semilandmarks were used to characterise the lateral contours of this part (Fig. 2b). For the membranous part, six type I landmarks were used to characterise the shape of the boundary between the stiff and membranous parts, and 16 equidistant semilandmarks were used to characterise the lateral contours (Fig. 2b). For the head, seven coplanar type II landmarks on the right side of the ventral view of the head were defined and collected; these landmarks included the anterocular, ocular, postocular and neck regions (Fig. 2c). For the pronotum, one coplanar type II landmark in the right anterolateral angle and 13 equidistant semilandmarks on the contour of the right side of the structure were defined and collected (Fig. 2d). The pronotum and head from individuals belonging to the Aguirre and Balbuena populations were not included in the analysis because only the forewings from these populations were available.

Semilandmarks were transformed to landmarks and analysed together with traditional landmarks [59]. We employed TPS DIG version 2.31 [60] to record landmarks and semilandmarks of the described structures. Data on the head, pronotum and forewing shape were extracted with a generalised full Procrustes fit and a projection to shape tangent space [61]. Procrustes coordinates as shape variables were used. Centroid size (CS; i.e. the square root of the sum of the squared distances from each landmark to the centroid of the configuration) was computed as a measure of size for each structure or part of the structure. These steps of morphometry analysis were performed using the free software MorphoJ version 1.07a [62]. Geometric variables of the forewing, portions of the forewing, the head and the pronotum were analysed separately. Variations across populations for the CS between Eastern and Western lineages for the forewing, part of the forewing, the head and the pronotum were explored, after testing for normality of the CS data with the Shapiro–Wilks, one-way ANOVA or Kruskal–Wallis tests using the software InfoStat version 2016 [63]. For shape measurements of the head, pronotum and forewings, we calculated Procrustes distances between lineages and evaluated the significance of these distances via a non-parametric test based on permutations (1000 runs) using MorphoJ [62]. We then performed discriminant function analysis (DFA) and calculated the percentage of phenotypic similarity between lineages using the cross-check test of discriminant analysis in InfoStat software [63]. Allometric relationships between shape and CS for the different structures or portions of the structure studied were checked using a multivariate regression of Procrustes coordinates on CS. The studied structures and the stiff portion of the wing showed no significant allometric effects according to the regression analyses. The membranous portion of the wing showed a significant allometric relationship (*P* = 0.0027), but with small allometric effects (2.30%).

## Results

### Divergence of* coI*

**Fig. 3:**
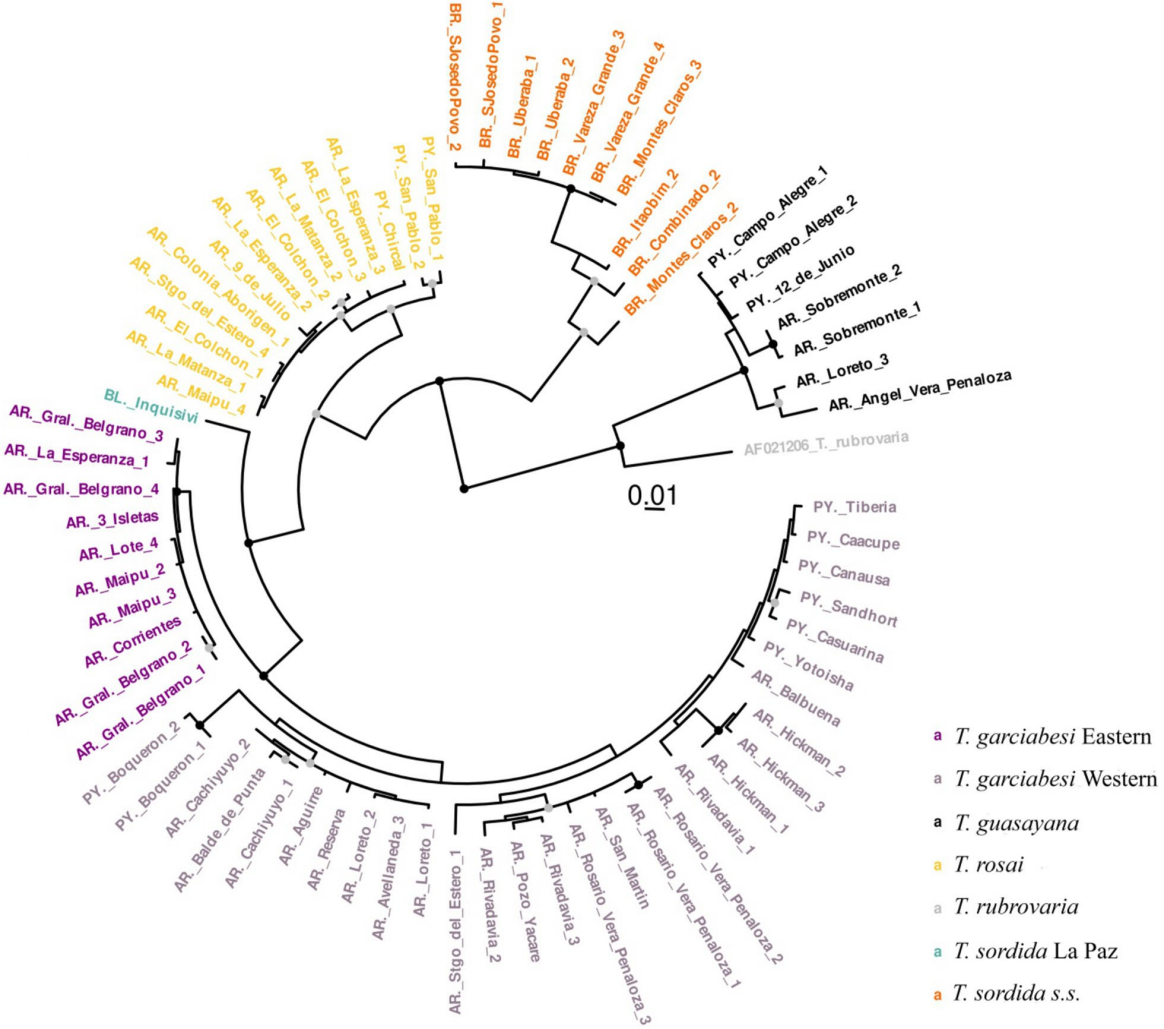
Maximum likelihood phylogenetic tree based on *coI* gene fragments for *Triatoma garciabesi*. *Triatoma rubroviaria*, *T. sordida* s.s, *T. rosai* and *T. sordida* from La Paz were included as outgroups. Tree topology reveals that *T. garciabesi* is composed of two clades, represented by Eastern and Western lineages, respectively. Grey- and black-filled circles above nodes represent statistical support obtained through bootstrap replications, 0.7 and 0.9, respectively. Letters included before the name of the population indicate country of origin: AR, Argentina; BL, Bolivia; BR, Brazil; PY, Paraguay.* coI*, Cytochrome* c* oxidase I gene; s.s., sensu stricto

The GTR + G + I substitution model was selected to construct the ML phylogenetic tree based on *coI* (Fig. 3). The tree shows five lineages within the ingroup, plus *T. rubrovaria* and *T. guasayana* individuals as outgroups, which are indicated in grey and black font in Fig. 3. The dendrogram drawn from the heatmap (Additional file 1: Figure S1) and the MDS plot (Additional file 2: Figure S2) reflect the same groupings as the ML tree. Mean distances within and between the lineages also indicate that lineages are well separated (Additional file 3: Table S1; Tables 2, 3). Average intraclade and interclade divergence ranged from 0.3% to 2.5% and from 3.2% to 16.3%, respectively. The greatest interspecific divergence was found between *T. garciabesi* Western lineage and other lineages, with the greatest divergence with *T. guasayana* (16.2%), followed by that with *T. sordida* sensu stricto (*T. sordida* s.s.; 12.6%), *T. rosai* (8.0%) and *T. sordida* La Paz (5.8%).

**Table 2:** Mean Kimura's two-parameter substitution model (K-2P)-based pairwise genetic distances between *Triatoma garciabesi* Eastern and Western lineages, and for the outgroups *Triatoma guasayana*, *T. rubrovaria*,* T. rosai* and *T. sordida* sensu stricto and *T. sordida* La Paz, for the *cytochrome c* *oxidase* *I* gene fragment

Groups	*T. garciabesi* Eastern	*T. garciabesi* Western	*T. guasayana*	*T. rubrovaria*	*T. rosai*	*T. sordida* LaPaz	*T. sordida* sensu stricto
*T. garciabesi* Eastern	0.0036	0.0325	0.1544	0.1368	0.0717	0.0497	0.1204
*T. garciabesi* Western	0.0325	0.0257	0.1617	0.1533	0.0805	0.0579	0.1257
*T. guasayana*	0.1544	0.1617	0.0198	0.0936	0.1474	0.1630	0.1429
*T. rubrovaria*	0.1368	0.1533	0.0936	NA	0.1339	0.1604	0.1475
*T. rosai*	0.0717	0.0805	0.1474	0.1339	0.0085	0.0632	0.1059
*T. sordida* LaPaz	0.0497	0.0579	0.1630	0.1604	0.0632	NA	0.0981
*T. sordida* sensu stricto	0.1204	0.1257	0.1429	0.1475	0.1058	0.0981	0.0188

Values in table are genetic distances calculated using the K-2P method for the *cytochrome c* *oxidase* *I *gene fragment

**Table 3:** Population genetic diversity indices and demographic history test estimated with cytochrome* c* oxidase I gene sequences for *T. garciabesi* Eastern and Western lineages, and for the outgroups *T. guasayana*,* T. rosai*,* T. rubrovaria* and *T. sordida* sensu stricto and *T. sordida* La Paz

Species	*N*	S	*N*h	*Hd*	*pi*	Tajima’s* D*	TajimaPval
*T. sordida* sensu stricto	10	40	9	0.9778	0.0202	− 0.5209	0.6024
*T. garciabesi* Eastern	10	7	8	0.9556	0.0034	− 0.5838	0.5593
*T. garciabesi* Western	29	69	28	0.9975	0.0248	− 0.5216	0.6019
*T. rosai*	14	23	14	1	0.009	− 0.9761	0.3290
*T. sordida* La Paz	1	0	1	NA	NA	NA	NA
*T. guasayana*	7	30	7	1	0.0193	− 0.1141	0.9092
*T. rubrovaria*	1	0	1	NA	NA	NA	NA

N, Number of individuals; S, segregating sites;* N*h, number of haplotypes; Hd, haplotype diversity; pi, nucleotide diversity; Tajima’s* D*, value of Tajima’s neutrality test; TajimaPval, *P*-value of Tajima’s neutrality test

**Fig. 4:**
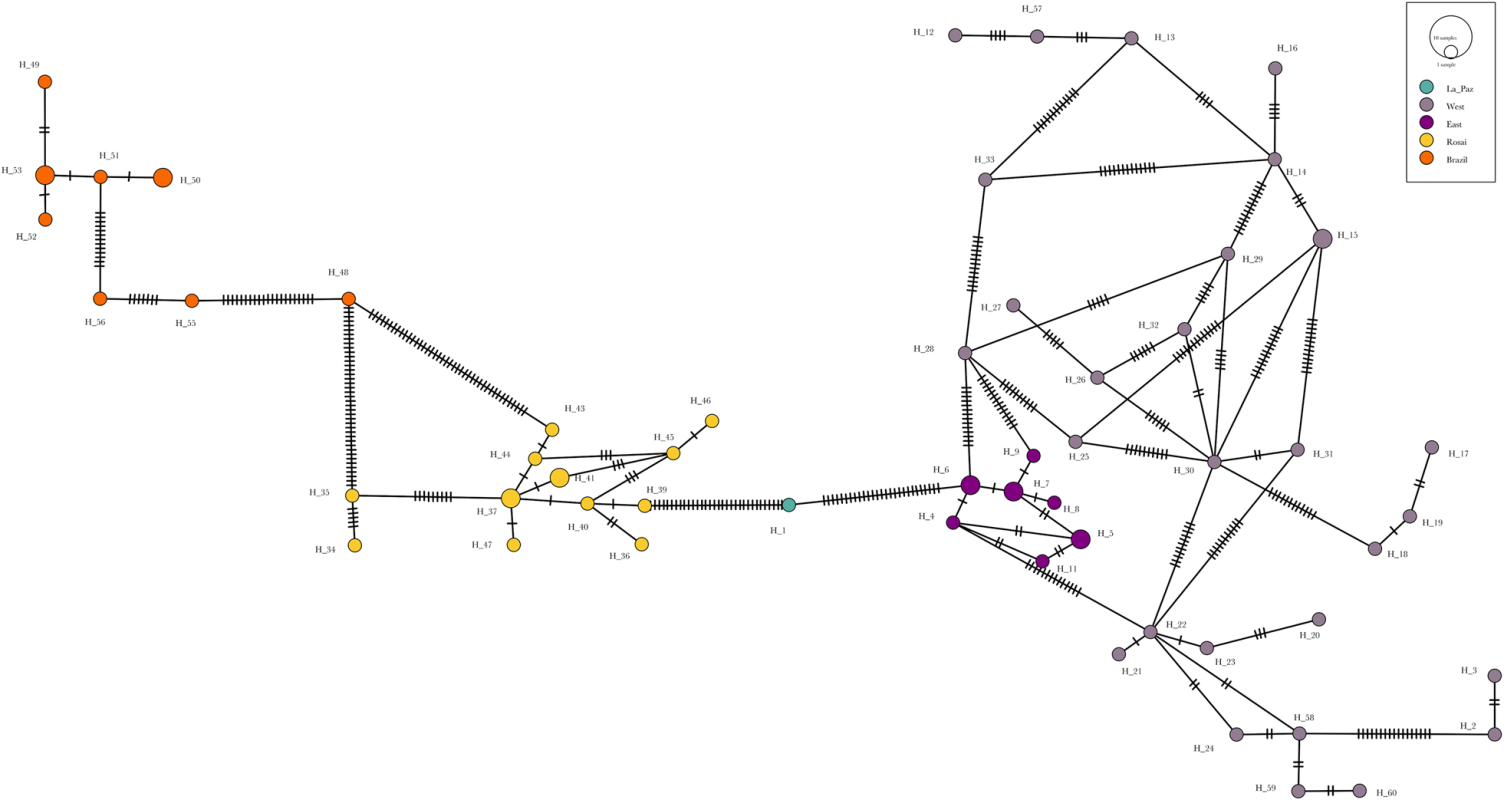
Minimum spanning network indicating the same clades as those of the maximum likelihood tree (Fig. 3). Nodes represent the haplotypes (see Fig. 3 for color coding), with their size proportional to the frequency of the haplotype. The numbers of mutational steps separating haplotypes are represented by the short vertical lines along each branch of the network. Individuals within haplotypes are depicted in Additional file 4: table S2.

As expected, the shortest pairwise genetic distance was observed between both *T. garciabesi* lineages (mean 3.2%, range 2.3–3.8%) (Additional file 3: Table S1; Tables 2, 3). Within *T. garciabesi*, the Western lineage showed the highest genetic distance among individuals, which also had the highest values for haplotype and nucleotide diversity (Table 3; Additional file 3: Table S1). The haplotype network also supports the delimitation of the lineages detected by genetic distance and on the tree topology. All lineages are separated by several mutation steps. Furthermore, the network shows the great diversity of the *T. garciabesi* Western lineage (Fig. 4).

### Forewing size, wing aspect ratio and size of the stiff and membranous portions of the forewing differed between *coI*-defined lineages

Forewing CS varied significantly between the Eastern and Western lineages (*F*_(2, 197)_ = 6.929, *P* = 0.0092), with forewings from the Eastern lineage being larger than those from the Western lineage (mean = 7.741, standard deviation [SD] = 0.475 vs mean = 7.543, SD = 0.468 for Eastern vs Western lineages, respectively). Wing aspect ratio also varied significantly between lineages (*F*_(2, 197)_ = 140.31, *P* < 0.0001), being bigger for the Western lineage (mean = 16.712, SD = 1.539 vs mean = 20.537, SD = 2.119 for Eastern vs Western lineages, respectively). The size of the stiff portion of the forewing for the Eastern lineage was significantly larger than that of the Western lineage (*F*_(2, 197)_ = 8.967, *P* = 0.0031) (mean = 9.442, SD = 0.708 vs mean = 9.125, SD = 0.633 for Eastern vs Western lineages, respectively). No significant differences between lineages were found for the size of the membranous portion (*F*_(2, 197)_ = 0.0555, *P* = 0.8141).

### Forewing shape differed between *coI*-defined lineages

**Fig. 5:**
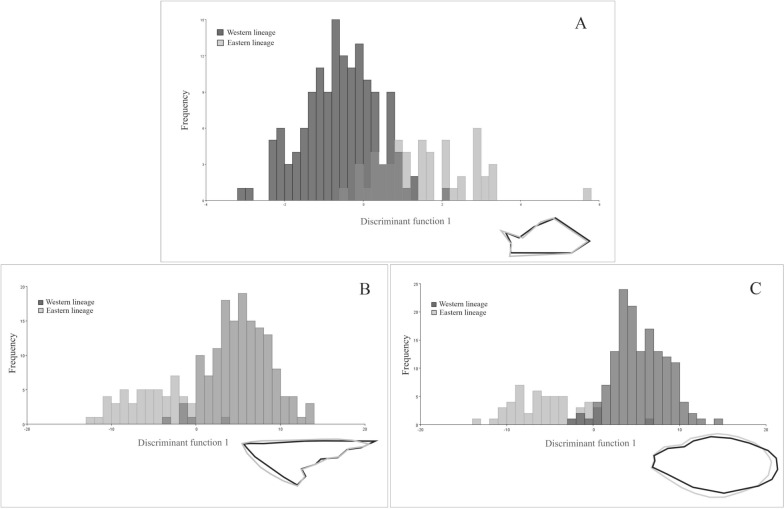
Frequency distribution of the first axis of the discriminant function analysis (DFA) for forewing shape (**a**) and for the stiff (**b**) and membranous (**c**) portions of the forewing for the Eastern and Western lineages of *T. garciabesi*. The shape changes associated with the first axis of the DFA are visualised as configurations corresponding to extreme positions of the axis. Black, configuration for negative extreme scores; grey, configuration for positive extreme scores

The first discriminant factor of the DFA explained 100% of the total variation. The Eastern and Western lineages were well differentiated along the first DFA axis (Fig. 5). Both Mahalanobis and Procrustes distances were significantly different between lineages (Mahalanobis distance = 2.172, *P*  < 0.0001; Procrustes distance = 0.0279, *P* < 0.0001,). Forewing shape changes, corresponding to extreme positions of the first axis of the DFA, involved all landmark configurations except for landmark 8. Changes in forewing shape in the Eastern lineage compared with the Western lineage involved a more developed stiff portion and a less developed membranous portion (Fig. 5a). Analysis of the forewings of the Eastern and Western lineages revealed 14.65% misclassified individuals; of all these misclassified individuals, 55.20% corresponded to the Eastern lineage and 44.8% to the Western lineage. When the populations with wrongly assigned individuals based on forewing shape were considered, ten populations (5 from the Eastern lineage and 5 from the Western lineage) had individuals assigned to the other lineage. Most of these populations (7 of 9 populations) were located at the distribution boundaries between the two lineages (Table 4; Fig. 1).

**Table 4:** Number of wrongly assigned individuals derived from discriminant function analysis for variation in *T. garciabesi* wing shape

*Triatoma garciabesi* lineage based on *coI*	Population	Number of individuals	Number (percentage) of wrongly assigned individuals	Number (percentage) of individuals assigned to the other *coI* group
Eastern	3 Isletas	8	6 (75%)	4 (66.67%)
Eastern	Corrientes	5	0 (0%)	0 (0%)
Eastern	Crucero Gral Belgrano	21	5 (23.8%)	3 (60%)
Eastern	La Esperanza	8	2 (25%)	1 (50%)
Eastern	Lote 4	5	1 (20%)	1 (100%)
Eastern	Maipú	6	2 (33.33%)	2 (100%)
Western	Aguirre	9	3 (33.33%)	1 (33.33%)
Western	Avellaneda	10	5 (50%)	1 (20%)
Western	Balbuena	9	2 (22%)	0 (0%)
Western	Balde de Punta	17	7 (41.18%)	0 (0%)
Western	Caacupé	5	3 (60%)	0 (0%)
Western	Canausa	5	3 (60%)	0 (0%)
Western	Casuarina	6	3 (50%)	0 (0%)
Western	Cruz del Eje	15	5 (53.33%)	0 (0%)
Western	Gral San Martín	9	5 (55.56%)	1 (20%)
Western	Hickman	5	4 (80%)	0 (0%)
Western	Loreto	5	4 (80%)	2 (50%)
Western	Pozo Yacaré	5	2 (40%)	0 (0%)
Western	Reserva Telteca	6	0 (0%)	0 (0%)
Western	Rivadavia	8	4 (50%)	0 (0%)
Western	Rosario Vera Peñaloza	10	5 (50%)	2 (60%)
Western	Sandhort	7	2 (28.57%)	0 (0%)
Western	Tiberia	7	5 (71.43%)	0 (0%)
Western	Yotoisha	7	3 (42.86%)	0 (0%)

*coI* *cytochrome c* *oxidase* *I* gene

### Stiff and membranous portions of the forewing differed between *coI-*defined lineages

The DFA for the shape of the contour of the stiff and membranous portions of the forewing showed that the Eastern and Western lineages were well differentiated (Fig. 5b, c). The first discriminant factor of both DFAs explained 100% of the total variation. Both Mahalanobis and Procrustes distances were significantly different between lineages for both portions of the forewing (stiff portion: Mahalanobis distance = 3.302, *P* < 0.0001 and Procrustes distance = 0.0271, *P* < 0.0001; membraneous portion: Mahalanobis distance = 3.285, *P* < 0.0001 and Procrustes distance = 0.0169, *P* < 0.0001). Changes in the shape of the contour of the stiff portion showed that the Eastern lineage had a wider shape than the Western lineage, mainly involving the basal and proximal parts of this portion of the forewing (Fig. 5b). For the shape of the contour of the membranous portion, the Western lineage exhibited a longer and narrower shape of the contour than the Eastern lineage (Fig. 5c).

### Pronotum size differed between *coI*-defined lineages

A Kruskal–Wallis’s test of the pronotum CS showed significant differences between the Eastern and Western lineages (*H* = 5.82, *P* = 0.016). Pronotum size was larger in individuals from the Eastern lineage than in those from the Western lineage (Dunn post-hoc test, *P* < 0.001) (mean = 1.508, SD = 0.090 vs mean = 1.488, SD = 0.116 for Eastern vs Western lineages, respectively).

### Pronotum shape differed between *coI*-defined lineages

**Fig. 6:**
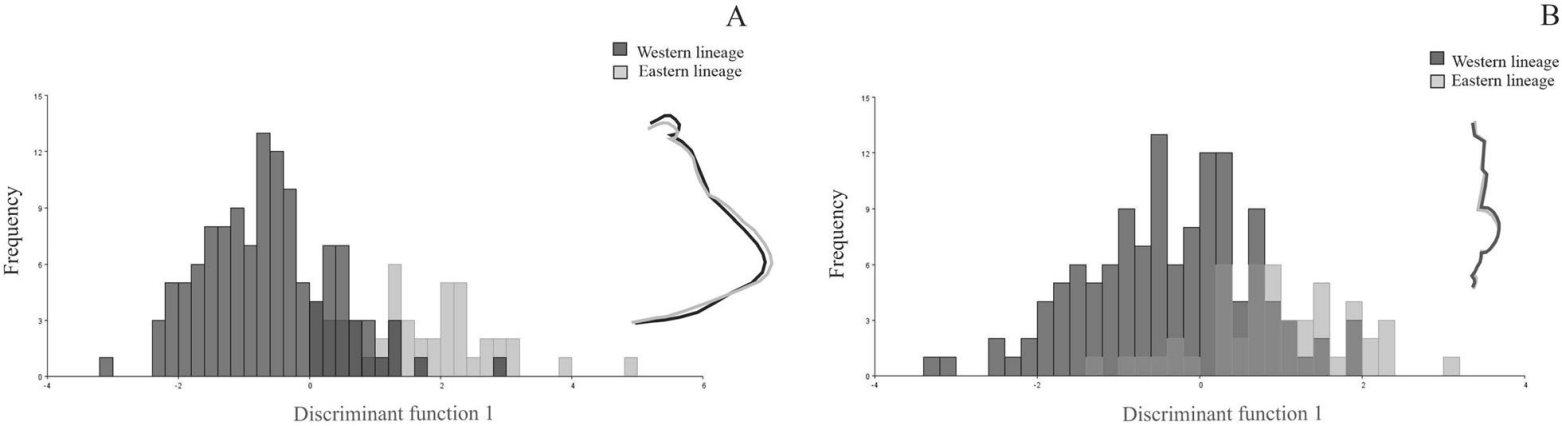
Frequency distribution of the first axis of the discriminant function analysis (DFA) for pronotum (**a**) and head (**b**) shape for *T. garciabesi* Eastern and Western lineages. The changes in shape associated with the first axis of the DFA are visualised as configurations corresponding to extreme positions of the axis. Black indicates the configuration for negative extreme scores; grey indicates the configuration for positive extreme scores

The first DFA axis accumulated 100% of the total variation for the forewing. Both lineages were defined in the space of the first DFA (Fig. 6a). Mahalanobis distance was significantly different between lineages (Mahalanobis distance = 2.253, *P* < 0.0001), whereas Procrustes distance was not significantly different between lineages (Procrustes distances = 0.0153, *P* = 0.0679). Changes in pronotum shape involved mostly landmarks located in the anterolateral angle and those located in the humeral angle (Fig. 6a). Analysis of pronotum shape in the Eastern and Western lineages showed 13.53% misclassified individuals. The analysis including the populations with wrongly assigned individuals showed that 16 populations (6 from the Eastern lineage and 10 from the Western lineage) were misclassified. Regarding pronotum shape, most of the populations that exhibited misclassified individuals were located at the border of the distribution area of both *coI* lineages (9 of 11 populations) (Table 5; Fig. 1).

**Table 5:** Number of wrongly assigned individuals derived from discriminant function analysis for variation in *T. garciabesi* pronotum shape

*Triatoma garciabesi* lineage based on *coI*	Population	Number of individuals	Number (percentage) of wrongly assigned individuals	Number (percentage) of individuals assigned to the other *coI* group
Eastern	3 Isletas	8	3 (37.5%)	1 (70.83%)
Eastern	Corrientes	5	1 (20%)	1 (100%)
Eastern	Crucero Gral Belgrano	17	7 (41.18%)	4 (57.14%)
Eastern	La Esperanza	8	5 (62.5%)	4 (80%)
Eastern	Lote 4	6	4 (66.67%)	3 (75%)
Eastern	Maipú	5	3 (60%)	2 (66.67%)
Western	Avellaneda	10	5 (50%)	(0%)
Western	Balde de Punta	17	14 (82.35%)	4 (28.57%)
Western	Caacupé	5	2 (40%)	1 (50%)
Western	Canausa	5	1 (20%)	0 (0%)
Western	Casuarina	6	4 (66.67%)	1 (25%)
Western	Cruz del Eje	16	13 (81.25%)	2 (15.38%)
Western	Gral San Martín	9	6 (66.67%)	1 (16.67%)
Western	Hickman	5	4 (80%)	1 (25%)
Western	Loreto	5	3 (60%)	1 (33.33%)
Western	Pozo Yacaré	5	3 (60%)	0 (0%)
Western	Reserva Telteca	6	0 (0%)	0 (0%)
Western	Rivadavia	8	4 (50%)	2 (50%)
Western	Rosario Vera Peñaloza	10	7 (70%)	2 (28.57%)
Western	Sandhort	7	3 (42.86%)	0 (0%)
Western	Tiberia	7	6 (85.7%)	0 (0%)
Western	Yotoisha	5	4 (80%)	2 (50%)

*coI* *cytochrome c* *oxidase* *I *gene

### Head shape-but not size-differed between *coI*-defined lineages

No significant differences in head CS between Eastern and Western lineages were observed (ANOVA, *F*_(2, 173)_ = 0.79, *P* = 0.3767). For wings, the first discriminant factor of the DFA explained 100% of the total variation. Eastern and Western lineages were differentiated along the first DFA axis (Fig. 6b). There were significant differences in both the Mahalanobis and Procrustes distances between lineages (Mahalanobis distance = 1.337, *P* < 0.0001; Procrustes distance = 0.0136, *P* < 0.0001). Changes in head shape between lineages involved anterocular, ocular and neck regions (Figs. 2, 6b). Heads of individuals from the Western lineages exhibited a widening of the anterior segment and greater eye development than heads of individuals of the Eastern lineages. DFA analysis of head shape in the Eastern and Western lineages showed 24.00% of misclassified individuals. When the populations with wrongly assigned individuals were included in the analysis, 16 populations (all populations from Eastern lineage and 10 from Western lineage) showed misclassified individuals (Table 6). Half of the populations (8 of 16 populations) that had misclassified individuals were located at the border of the distribution area of both *coI* lineages.

**Table 6:** Number of wrongly assigned individuals derived from discriminant function analysis for variation in *T. garciabesi* head shape

*Triatoma garciabesi* lineage based on *coI*	Population	Number of individuals	Number (percentage) of wrongly assigned individuals	Number (percentage) of individuals assigned to the other *coI* group
Eastern	3 Isletas	8	4 (50%)	3 (75%)
Eastern	Corrientes	5	1 (20%)	1 (100%)
Eastern	Crucero Gral Belgrano	17	7 (41.18%)	4 (57.14%)
Eastern	La Esperanza	8	5 (62.5%)	4 (80%)
Eastern	Lote 4	6	4 (66.67%)	3 (75%)
Eastern	Maipú	5	3 (60%)	2 (66.67%)
Western	Avellaneda	10	5 (50%)	0 (0%)
Western	Balde de Punta	17	14 (82.35%)	4 (28.57%)
Western	Caacupé	5	2 (40%)	1 (50%)
Western	Canausa	5	1 (20%)	0 (0%)
Western	Casuarina	6	4 (66.67%)	1 (25%)
Western	Cruz del Eje	16	13 (81.25%)	2 (15.38%)
Western	Gral San Martín	9	6 (66.67%)	1 (16.67%)
Western	Hickman	5	4 (80%)	1 (25%)
Western	Loreto	5	3 (60%)	1 (33.33%)
Western	Pozo Yacaré	5	3 (60%)	0 (0%)
Western	Reserva Telteca	6	0 (0%)	0 (0%)
Western	Rivadavia	8	4 (50%)	2 (50%)
Western	Rosario Vera Peñaloza	10	7 (70%)	2 (28.57%)
Western	Sandhort	7	3 (42.86%)	0 (0%)
Western	Tiberia	7	6 (85.71%)	0 (0%)
Western	Yotoisha	5	4 (80%)	2 (50%)

*coI* *cytochrome c* *oxidase* *I* gene

## Discussion

### Genetic divergence within T. garciabesi

The results obtained in this study demonstrate the existence of two separate lineages within *T. garciabesi*, here referred to as the Eastern and Western lineages. The lineages showed a comparatively different non-overlapping geographical distribution, with the Eastern lineage being restricted to and clustered in a part of the centre and east of the Argentine provinces of Chaco and Formosa, which coincides with the Humid Chaco ecoregion (there was only 1 population at the boundary between the Humid and the Dry Chaco and 1 population located in the Paraná Flooded Savanna; Fig. 1). The Western lineage is present throughout the rest of the species distribution range. Most populations are located in the Dry Chaco ecoregion, and two populations are located in the High and Low Monte ecoregions. The Dry Chaco ecoregion exhibits a marked longitudinal precipitation gradient from east to west and marked differences in phytogeographical characteristics [48], suggesting that lineage diversification could be associated with landscape characteristics.

Studies using mitochondrial genes (*cytb*, *coI* and *coII*) for phylogenetic separation of closely related species have been carried out in other triatomine species (for review see [4]), such as, for example, *Rhodnius prolixus* Stål, 1859 and *Rhodnius robustus* Larrousse, 1927 sensu lato [64], *Triatoma rubida* Uhler, 1894 and *Triatoma recurva* Stål, 1868 [65], species of the *Triatoma brasiliensis* complex [66] and species of the *Mepraia* genus [67]. These genes have also been used in population analyses of *Triatoma sanguisuga* Leconte, 1855 [68] and *T. infestans* [69]. In the present study, the inference of the phylogenetic tree constructed using the ML method allowed us to classify the samples into seven clades; thus, the populations were separated into: *T. garciabesi* from the Eastern lineage, *T. garciabesi* from the Western lineage, *T. guasayana*, *T. rosai*, *T. rubrovaria*, *T. sordida* La Paz and *T. sordida* s.s. The calculation of *coI* gene pairwise distance is generally used to predict cryptic or novel species ([28] and references cited therein). In general, intraspecific genetic diversity in mitochondrial genes is rarely > 2%, and mostly < 1% [27, 70, 71]. Meier et al. [70] indicate that if the objective is to predict cryptic species based on genetic distance, the smallest interspecific distance has to be used. If the minimum interspecific genetic value distance from congener species is ≥ 2%, it is possible to avoid overestimating species diversity based on empirical thresholds. However, in studies on triatomines, different genes and regions within the genes have been used. Therefore, different consensuses were taken into account to decide whether the variation belonged to different species or to populations within the same species. For example, in one study, the mean pairwise distance between species for the *coI* sequence was about 7% (e.g. *Mepraia gajardoi* Frias, Henry & Gonzalez, 1998 and *M. spinolai* [67]). For the genus *Triatoma*, the largest mean pairwise nucleotide distance was found to be 5.2% between North and Centre-South *Triatoma patagonica* Del Ponte, 1929 lineages [72]. Similar values between pairs of closely related species (*T. infestans–Triatoma platensis* Neiva 1913 and *T. sordida–T. garciabesi*) have been reported for other mitochondrial gene fragments (*cytb*) [66]. For two *T. brasiliensis* populations, the pairwise distance of > 8.4% found between them was one of the arguments that supported their placement to the species level. Our results for *T. garciabesi* lineages showed a mean pairwise distance between the Eastern and Western lineages of 3.2%. Hence, the *coI* genetic distances observed for *T. garciabesi* lineages would not be sufficient to consider them as distinct species. However, the environmental conditions to which lineages are exposed would play a crucial role in increasing genetic differentiation through divergent selection, a pattern known as isolation-by-environment [73, 74].

### Congruence between genetic and morphometric variation

In agreement with the diversity that has been observed in *coI*, our results showed morphometry differentiation between the two *T. garciabesi* lineages in the forewing, head and pronotum measurements. Patterns of variations in size or shape have been interpreted previously in terms of their evolutionary implications [75]. It is known that environmental factors (e.g. temperature and humidity) determine phenotypic characteristics in several triatomine species (reviewed in [76]). Size differentiation of morphological attributes has been suggested to be strongly (but not exclusively) influenced by the ecological characteristics of species and populations [76]. If we consider that genetic and historical attributes are often thought to affect shape variation of morphological attributes [77], we can assume that the consistent size differentiation observed in the studied morphological attributes of *T. garciabesi* lineages is due to ecological differences between the ecoregions where the *T. garciabesi* Eastern and Western lineages occur. Individuals from the Eastern lineage showed a greater size, both of heads and pronota, than individuals from the Western lineage. In other triatomine species, forewing size has been found to be a good predictor of body length [78]. Moreover, total body length has been found to be closely correlated with total body weight and with female fecundity [79]. These results may indicate that genetic variability within populations of *T. garciabesi* plays a role in size variation in these populations. However, this is a preliminary suggestion, and it may be necessary to carry out additional analyses, experiments or studies to confirm and better understand this association between genetic variability and size variation. Differences in shape variation between lineages suggest genetic variation. Interestingly, the discriminant analysis of shape differentiation showed higher percentages of misclassified individuals in populations located at the distribution boundaries of both clades than in the remaining populations. This pattern was observed mainly in the shape of forewings and pronotum, suggesting the presence of a transition zone or ecotone where the morphological characteristics are in transition between the two lineages. This could be an area of hybridisation or a zone of genetic contact. Phenotypic diversity is often known to be higher at habitat boundaries than in more homogeneous interior regions of the habitats [80]. Our results also suggest adaptive divergence associated with habitat characteristics, but the degree of reproductive isolation of the two lineages is still unknown. The mechanisms that limit gene flow between populations can be studied by making experimental crosses and analysing the offspring [81]. Further studies to elucidate a possible reproductive isolation between the lineages may help to understand whether genetic differentiation results in some level of reproductive isolation.

The association between genetic and morphological variation has been studied previously in triatomines [25, 72, 82, 83], providing data that improve current understanding of the different speciation processes that may be operating in this subfamily. For *Rhodnius pallescens* Barber 1932 lineages, diversification was found to be mainly influenced by biogeographical events [80], while for *T. sordida*, the differentiation between populations from eastern and western regions of Paraguay was suggested by the authors of the study to be associated with eco-geographical isolation by distance [83]. In *T. patagonica*, genetic and morphological diversification were explained by an ecological diversification accompanied with cytogenetic differentiation and partial reproductive isolation of the most distant populations [72].

### Implications of variation in flight-related traits and mitochondrial lineage distribution

Dispersal abilities are of major importance in establishing and rearranging the geographical distribution of species [29]. The *coI* mitochondrial gene, as all protein coding genes in the mitochondrial genome, is considered to be under purifying selection, since it constantly sweeps away deleterious nonsynonymous mutations [84]. On the other hand, morphometry of flight-related traits is more likely to have evolved under adaptative selection. In particular, the forewing parts of the hemelytra are expected to be under strong selective pressure [36]. The morphometry characteristics of these flight-related traits and the parts of the hemelytra of this species suggest which forewing morphotype is selected in each lineage in relation to the environment where they develop. These characteristics would determine the type of flight of each lineage, which would improve our understanding of how flight-related traits shape the geographical distribution of genetic diversity in this species. Long and narrow forewings give greater deformation capacity at a lower energy cost for long distance flights. Despite this assertion, no specific tests of flight capacity have been carried out in triatomines. While previous studies on other insects may provide indirect support, the morphology of triatomine hemelytra is different and this type of study was necessary to properly interpret these results.

The aspect ratio indicates the flight capacity, with higher values of this ratio being compatible with more efficient and longer flights [38, 85]. In dragonflies (Order: Odonata), the functional roles of forewing structural components suggest that forewings require a balance between flexibility and stiffness [86]. Our results showed that the portions of the forewings of the two lineages of *T. garciabesi* showed differences in both shape and aspect ratio. The forewing of the Eastern lineage was larger than that of the Western lineage; however, when variation was broken down into the stiff and membranous portions, only the stiff portion was larger in the Eastern lineage while the membranous portion did not differ between lineages. The analysis of shape variation showed that the stiff portion of individuals of the Eastern lineage is wider than that those of the Western lineage, whereas the membranous part of the Western lineage is longer and narrower than that of the Eastern lineage. The aspect ratio varied between lineages, with individuals of the Western lineage exhibiting higher values.

The morphological differences observed in the hemelytra of the two lineages suggest different types of flight. The Western lineage would have more aerodynamically stable, less energetically costly and longer flights. The morphological attributes of the Eastern lineage are compatible with short, slow and somewhat unstable flights. Hence, these morphological attributes would determine different types of flight associated with the lineages. Among the characteristics that define the Humid Chaco ecoregion, in addition to the abundant rainfall, which favours vegetation diversity, there is little thermal amplitude, and the local fauna is abundant [47, 87]. These characteristics would allow a long time window for the development of triatomine populations; similarly, the great availability of local fauna means a reduced need to disperse in search of hosts. Thus, the forewing attributes corresponding to short and agile flights could be an adaptation of individuals from the Humid Chaco region that would allow short and agile flights, and a consequent increased allocation of resources to reproduction. On the other hand, in the arid Dry Chaco and Monte ecoregions, the landscape is less lush and host availability is lower than in the Humid Chaco. These landscape characteristics would require longer and aerodynamically more stable flights, which would favour the search for hosts. It should be emphasised here that this study does not directly measure flight capacity and that these suggestions are based on morphological characteristics and extrapolated to the potential of the flight behaviours of this triatomine species. Dispersal capacity experiments would be necessary to validate and strengthen these statements.

Phenotypic variation in head shape also differed between lineages. Heads from the Western lineages exhibited a widening of the anterior segment and greater eye development than those of the Eastern lineages. These lineage-related changes in head conformation are consistent with the changes in forewing characteristics described above. In *M. spinolai*, macropteran males were found to have a greater convexity of the compound eyes and a greater interocular distance than micropteran males, which could imply that the former have a greater navigational capacity and sense of orientation during flight [41]. The characteristics of the forewings and heads help to understand the possible type of flight observed in each lineage. The types of flight are favoured by selective pressures linked to the landscape in which the lineage evolved. The pronotum does not follow the expected shape changes in relation to these characteristics, since in the Eastern lineage, the pronotum exhibits a greater development of the humeral angle, which suggests a greater development of the alar muscle.

In the present study, we were unable to molecularly characterise all individuals and assumed that all individuals from the same population belonged to the same species as when more than one individual from the same population were molecularly characterised, they were found to belong to the same species. This limitation does not undermine our conclusions since the pronounced genetic divergence is consistent with morphometry variation.

## Conclusions

Our results provide preliminary insights into the evolution and diversification of *T. garciabesi*. There is a pronounced *coI* mtDNA divergence, with the existence of two lineages with different distribution ranges. Consistent with genetic divergence, morphometry variation was clustered according to lineages, with congruent variation in size and shape of the forewing, pronotum and head.

### Supplementary Information

**Additional file 1: Figure S1.** Heatmap representation of pairwise genetic distances obtained for *coI* gene fragments for *T. garciabesi*, *T. rubroviaria*, *T. sordida* s.s, *T. rosai* and *T. sordida* from La Paz, calculated under the k-2p substitution model.

**Additional file 2: Figure S2.** Graphical representation of the multidimensional scaling analysis (MDS) performed for genetic distance of *coI* gene fragments of *T. garciabesi*, *T. rubroviaria*, *T. sordida* s.s,* T. rosai* and *T. sordida* from La Paz.

**Additional file 3: Table S1.** Pairwise k-2p genetic distances obtained for *coI* gene fragments, depicted for each individual.

**Additional file 4: Table S2.** Haplotype composition by frequency, lineage and individuals.

## Data Availability

The datasets supporting the conclusions of this article are included in the article and its additional files. Raw data are available from the corresponding author on reasonable request.

## References

[1] Chagas C (1909). Nova tripanossomíase humana: Estudos sobre a morfologia e o ciclo evolutivo do *Schizotrypanum cruzi* n. gen., n. sp., agente etiológico de nova entidade mórbida do homem. Mem Inst Oswaldo Cruz.

[2] WHO. Chagas disease. 2023. https://www.who.int/health-topics/chagas-disease. Accessed Sep 2023.

[3] Lent H, Wygodzinsky P (1979). Revision of the Triatominae (Hemiptera: Reduviidae) and their significance as vectors of Chagas disease. Bull Am Mus Nat Hist.

[4] Monteiro FA, Weirauch C, Felix M, Lazoski C, Abad-Franch F (2018). Evolution, systematics, and biogeography of the Triatominae, vectors of Chagas disease. Adv Parasitol.

[5] Schofield CJ, Galvão C (2009). Classification, evolution, and species groups within the Triatominae. Acta Trop.

[6] Pita S, Lorite P, Nattero J, Galvão C, Alevi KCC, Teves SC (2016). New arrangements on several species subcomplexes of *Triatoma* genus based on the chromosomal position of ribosomal genes (Hemiptera - Triatominae). Infect Genet Evol.

[7] Panzera F, Pita S, Nattero J, Panzera Y, Galvão C, Chavez T (2015). Cryptic speciation in the *Triatoma sordida* subcomplex (Hemiptera, Reduviidae) revealed by chromosomal markers. Parasit Vectors.

[8] Belintani T, Oliveira J, Pinotti H, Silva LA, Alevi KCC, Galvão C (2020). Phylogenetic and phenotypic relationships of the *Triatoma sordida* subcomplex (Hemiptera: Reduviidae: Triatominae). Acta Trop.

[9] Moriconi DE, Macedo-Lopes C, Sartorio A, Juárez MP, Girotti JR, Calderón-Fernández GM (2022). Chemotaxonomy of Five South American species of the *Triatoma* genus (Hemiptera: Reduviidae: Triatominae) based on their cuticle hydrocarbon pattern. J Med Entomol.

[10] Carcavallo RU, Cichero JA, Martínez A, Prosen F, Ronderos R (1967). Una nueva especie del gênero *Triatoma* Laporte (Hemiptera, Reduviidae, Triatominae). Segundas Jornadas Entomoepidemiológicas Argentinas.

[11] Jurberg J, Galvão C, Lent H, Monteiro F, Lopes CM, Panzera F (1998). Revalidação de *Triatoma garciabesi* Carcavallo, Cichero, Martínez, Prosen & Ronderos, 1967 (Hemiptera, Reduviidae). Entomol Vect.

[12] Carcavallo RU, de Casas SIC, Sherlock IA, Galíndez-Girón I, Jurberg J, Lent H, et al. Geographical distribution and alti-latitudinal dispersion. In: Carcavallo RU, Galíndez-Girón I, Jurberg J, Lent H, et al., editors. Atlas of Chagas’ disease vectors in the Americas, vol. III. Rio de Janeiro: Editora Fiocruz; 1999. p. 747–92.

[13] Gorla DE, Jurberg J, Catalá SS, Schofield CJ (1993). Systematics of *Triatoma sordida*, *T. guasayana* and *T. patagonica* (Hemiptera, Reduviidae). Mem Inst Oswaldo Cruz.

[14] Belintani T, Oliveira J, Pinotti H, Alevi KCC, Nascimento JD, Sasso-Cerri E (2021). Characterization of female external genitalia and eggs of four South American species of the *Triatoma* Laporte, 1832 Genus (Hemiptera: Reduviidae: Triatominae). Insects.

[15] Gurgel-Gonçalves R, Ferreira JBC, Rosa AF, Bar ME, Galvão C (2011). Geometric morphometrics and ecological niche modelling for delimitation of near-sibling triatomine species. Med Vet Entomol.

[16] Nattero J, Piccinali RV, Lopes CM, Hernández ML, Abahan L, Lobbia PA (2017). Morphometric variability among the species of the Sordida subcomplex (Hemiptera: Reduviidae: Triatominae): evidence for differentiation across the distribution range of *Triatoma sordida*. Parasit Vectors.

[17] Noireau F, Gutierrez T, Zegarra M, Flores R, Breniere SF, Cardozo L (1998). Cryptic speciation in *Triatoma sordida* (Hemiptera: Reduviidae) from the Bolivian Chaco. Trop Med Int Health.

[18] Justi SA, Russo CAM, Mallet JRS, Obara MT, Galvão C (2014). Molecular phylogeny of Triatomine (Hemiptera: Reduviidae: Triatominae). Parasit Vectors.

[19] Canale DM, Cecere MC, Chuit R, Gürtler RE (2000). Peridomestic distribution of *Triatoma garciabesi* and *Triatoma guasayana* in north-west Argentina. Med Vet Entomol.

[20] Cavallo MJ, Amelotti I, Gorla DE (2016). Invasion of rural houses by wild Triatominae in the Arid Chaco. J Vector Ecol.

[21] Cardozo M, Fiad FG, Crocco LB, Gorla DE (2021). Effect of habitat fragmentation on rural house invasion by sylvatic triatomines: a multiple landscape-scale approach. PLoS Negl Trop Dis.

[22] Carbajal-de-la-Fuente AL, Fernández MDP, Piccinali RV, Rodríguez-Planes LI, Duarte R, Gürtler RE (2019). Occurrence of domestic and intrusive triatomines (Hemiptera: Reduviidae) in sylvatic habitats of the temperate Monte Desert ecoregion of Argentina. Acta Trop.

[23] Cardozo M, Fiad FG, Crocco LB, Gorla DE (2021). Triatominae of the semi-arid Chaco in central Argentina. Acta Trop.

[24] Ceccarelli S, Balsalobre A, Cano ME, Canale D, Lobbia P, Stariolo R (2020). Analysis of Chagas disease vectors occurrence data: the Argentinean triatomine species database. Bio Data J.

[25] Kamimura EH, Viana MC, Lilioso M, Fontes FH, Pires-Silva D, Valença-Barbosa C (2020). Drivers of molecular and morphometric variation in *Triatoma brasiliensis* (Hemiptera: Triatominae): the resolution of geometric morphometrics for populational structuring on a microgeographical scale. Parasit Vectors.

[26] Filée J, Merle M, Bastide H, Mougel F, Bérenger JM, Folly-Ramos E (2022). Phylogenomics for Chagas disease vectors of the *Rhodnius* genus (Hemiptera, Triatominae): what we learn from mito-nuclear conflicts and recommendations. Front Ecol Evol.

[27] Moritz C, Dowling TE, Brown WM (1987). Evolution of animal mitochondrial DNA: relevance for population biology and systematics. Ann Rev Ecol System.

[28] Zhang H, Bu W (2022). Exploring large-scale patterns of genetic variation in the COI gene among Insecta: implications for DNA barcoding and threshold-based species delimitation studies. Insects.

[29] Renault D (2020). A review of the phenotypic traits associated with insect dispersal polymorphism, and experimental designs for sorting out resident and disperser phenotypes. Insects.

[30] Turlure C, Schtickzelle N, Van Dyck H, Seymoure B, Rutowski R (2016). Flight morphology, compound eye structure and dispersal in the bog and the cranberry fritillary butterflies: an inter- and intraspecific comparison. PLoS ONE.

[31] Dudley R (2000). The biomechanics of insect flight: form, function, evolution.

[32] Wisnivesky-Colli C, Gurtler RE, Solarz ND, Schweigmann NJ, Pietrokovsky SM, Alberti A (1993). Dispersive flight and house invasion by *Triatoma guasayana* and *Triatoma sordida* in Argentina. Mem Inst Oswaldo Cruz.

[33] Galvão C, Da Silva Rocha D, Jurberg J, Carcavallo R (2001). Início da atividade de vôo en *Triatoma infestans* (Klug 1834) e T melanosoma Martínez, Olmedo and Carcavallo, 1987 (Hemiptera, Reduviidae). Mem Inst Oswaldo Cruz.

[34] Schofield CJ, Lehane MJ, McEwen P, Catala SS, Gorla DE (1992). Dispersive flight by *Triatoma infestans* under natural climatic conditions in Argentina. Med Vet Entomol.

[35] Schweigmann N, Vallve S, Muscio O, Ghillini N, Alberti A, Wisnivesky-Colli C (1988). Dispersal flight by *Triatoma infestans* in an arid area of Argentina. Med Vet Entomol.

[36] Wootton RJ, Schaefer CW (1996). Functional wing morphology in Hemiptera systematics. Studies in Hemipteran phylogeny.

[37] DeVries PJ, Penz CM, Hill RI (2010). Vertical distribution, flight behaviour and evolution of wing morphology in Morpho butterflies. J Anim Ecol.

[38] Outomuro D, Adams DC, Johansson F (2013). Wing shape allometry and aerodynamics in calopterygid damselflies: a comparative approach. BMC Evol Biol.

[39] Fiad FG, Cardozo M, Rodríguez CS, Hernández ML, Crocco LB, Gorla DE (2022). Ecomorphological variation of the *Triatoma guasayana* wing shape in semi-arid Chaco region. Acta Trop.

[40] Betts CR, Wootton RJ (1988). Wing shape and flight behaviour in butterflies (Lepidoptera: Papilionoidea and Hesperioidea): a preliminary analysis. J Exp Biol.

[41] Hernández ML, Dujardin JP, Gorla DE, Catalá SS (2015). Can body traits, other than wings, reflect the flight ability of Triatominae bugs?. Rev Soc Bra Med Trop.

[42] Hernández ML, Espinoza J, Gomez M, Gorla D (2020). Morphological changes associated with brachypterous *Triatoma guasayana* (Hemiptera, Reduviidae) and their relationship with flight. Int J Trop Insect Sci.

[43] Gigena GV, Rodríguez CS, Fiad FG, Hernández ML, Carbajal-de-la-Fuente AL, Piccinali RV (2023). Phenotypic variability in traits related to flight dispersal in the wing dimorphic species *Triatoma guasayana*. Parasit Vectors.

[44] Almeida CE, Oliveira HL, Correia N, Dornak LL, Gumiel M, Neiva VL (2012). Dispersion capacity of *Triatoma sherlocki*, *Triatoma juazeirensis* and laboratory-bred hybrids. Acta Trop.

[45] Cote J, Clobert J, Fitze PS (2007). Mother-offspring competition promotes colonization success. Proc Natl Acad Sci USA.

[46] Olson DM, Dinerstein E, Wikramanayake ED, Burgess ND, Powell GVN, Underwood EC (2001). Terrestrial ecoregions of the world: a new map of life on Earth. Bioscience.

[47] Ginzburg R, Adámoli J, Brown A, Martínez Ortíz U, Acerbi M, Corcuera J (2005). Situación ambiental en el Chaco Húmedo. La Situación Ambiental Argentina.

[48] Apodaca MJ, Crisci JV, Katinas L. Las provincias fitogeográficas de la República Argentina: definición y sus principales áreas protegidas. In: Casas RR, Albarracín GF, editors. El deterioro de los suelos y del ambiente en la Argentina. Argentina: Fundación para la Educación, la Ciencia y la Cultura. 2015. p. 79–101

[49] Baldwin JD, Bass AL, Bowen BW, Clark WH (1998). Molecular phylogeny and biogeography of the marine shrimp *Penaeus*. Mol Phylogenet Evol.

[50] Palumbi SR, Benzie J (1991). Large mitochondrial DNA differences between morphologically similar penaeid shrimp. Mol Mar Biol Biotechnol.

[51] Katoh K, Standley DM (2013). MAFFT multiple sequence alignment software version 7: improvements in performance and usability. Mol Biol Evol.

[52] Schliep KP (2011). phangorn: phylogenetic analysis in R. Bioinformatics.

[53] Yu G, Smith DK, Zhu H, Guan Y, Lam TT-Y (2017). GGTREE: an R package for visualization and annotation of phylogenetic trees with their covariates and other associated data. Methods Ecol Evol.

[54] Paradis E, Schliep K (2019). ape 5.0: an environment for modern phylogenetics and evolutionary analyses in R. Bioinformatics.

[55] Paradis E (2010). pegas: an R package for population genetics with an integrated-modular approach. Bioinformatics.

[56] Cox TF, Cox MAA (2001). Mutidimensional scaling.

[57] Leigh JW, Bryant D (2015). popart: full-feature software for haplotype network construction. Methods Ecol Evol.

[58] Bookstein FL (1991). Morphometric tools for landmark data geometric and biology.

[59] Gunz P, Mitteroecker P (2013). Semilandmarks: a method for quantifying curves and surfaces. Hystrix.

[60] Rohlf FJ. TpsDig, digitize landmarks and outlines version 2.0. New York: Department of ecology and evolution State University of New York at Stony Brook; 2004.

[61] Rohlf FJ (1999). Shape statistics: procrustes superimpositions and tangent spaces. J Class.

[62] Klingenberg CP (2011). MorphoJ: an integrated software package for geometric morphometrics. Mol Ecol Resour.

[63] Di Rienzo JA, Casanoves F, Balzarini MG, Gonzalez L, Tablada M, Robledo CW (2016). InfoStat, versión 2016.

[64] Monteiro FA, Barrett TV, Fitzpatrick S, Cordón-Rosales C, Feliciangeli D, Beard CB (2003). Molecular phylogeography of the Amazonian Chagas disease vectors *Rhodnius prolixus* and *R. robustus*. Mol Ecol.

[65] Pfeiler E, Bitler BG, Ramsey JM, Palacios-Cardiel C, Markow TA (2006). Genetic variation, population structure, and phylogenetic relationships of *Triatoma rubida* and *T. recurva* (Hemiptera: Reduviidae: Triatominae) from the Sonoran Desert, insect vectors of the Chagas’ disease parasite *Trypanosoma cruzi*. Mol Phylogenet Evol..

[66] Monteiro FA, Donnelly MJ, Beard CB, Costa J (2004). Nested clade and phylogeographic analyses of the Chagas disease vector *Triatoma brasiliensis* in Northeast Brazil. Mol Phylogenet Evol.

[67] Calleros L, Panzera F, Bargues MD, Monteiro FA, Klisiowicz DR, Zuriaga MA (2020). Systematics of *Mepraia* (Hemiptera-Reduviidae): cytogenetic and molecular variation. Infect Genet Evol.

[68] De La Rua N, Stevens L, Dorn PL (2011). High genetic diversity in a single population of *Triatoma sanguisuga* (LeConte, 1855) inferred from two mitochondrial markers: cytochrome b and 16S ribosomal DNA. Infect Genet Evol.

[69] Ceballos LA, Piccinali RV, Berkunsky I, Kitron U, Gürtler RE (2009). First finding of melanic sylvatic *Triatoma infestans* (Hemiptera: Reduviidae) colonies in the Argentine Chaco. J Med Entomol.

[70] Meier R, Shiyang K, Vaidya G, Ng PKL (2006). DNA barcoding and taxonomy in Diptera: a tale of high intraspecific variability and low identification success. Syst Biol.

[71] Avise JC (2000). Phylogeography: the history and formation of species.

[72] Nattero J, Pita S, Calleros L, Crocco L, Panzera Y, Rodríguez CS (2016). Morphological and genetic differentiation within the Southernmost vector of Chagas disease: *Triatoma patagonica* (Hemiptera – Reduviidae). PLoS ONE.

[73] Wang IJ, Summers K (2010). Genetic structure is correlated with phenotypic divergence rather than geographic isolation in the highly polymorphic strawberry poison-dart frog. Mol Ecol.

[74] Sexton JP, Hangartner SB, Hoffmann AA (2014). Genetic isolation by environment or distance: which pattern of gene flow is most common?. Evolution.

[75] Pigliucci M (2005). Evolution of phenotypic plasticity: where are we going now?. Trends Ecol Evol.

[76] Dujardin JP, Costa J, Bustamante D, Jaramillo N, Catalá S (2009). Deciphering morphology in Triatominae: the evolutionary signals. Acta Trop.

[77] Dujardin JP, Slice D, Tibayrenc M (2007). Geometric morphometrics. Contributions to medical entomology. Encyclopedia of infectious diseases. Modern methodologies.

[78] Gaspe MS, Schachter-Broide J, Gurevitz JM, Kitron U, Gürtler RE, Dujardin JP (2012). Microgeographic spatial structuring of *Triatoma infestans* (Hemiptera: Reduviidae) populations using wing geometric morphometry in the Argentine Chaco. J Med Entomol.

[79] Gürtler RE, Fernández MDP, Cecere MC, Cohen JE (2017). Body size and hosts of *Triatoma infestans* populations affect the size of bloodmeal contents and female fecundity in rural northwestern Argentina. PLoS Negl Trop Dis.

[80] Schilthuizen M (2000). Ecotone: speciation-prone. Trends Ecol Evol.

[81] Arnold M (1997). Natural hybridization and evolution.

[82] Díaz S, Francisco P, Jaramillo ON, Ruben P, Fernández R (2014). Gnetic, cytogenetic and morphological trends in the evolution of the *Rhodnius* (Triatominae: Rhodniini) Trans-Andean Group. PLoS ONE.

[83] González-Brítez NE, Carrasco HJ, Martínez PCE, Feliciangeli MD, Maldonado M, López E (2014). Genetic and morphometric variability of *Triatoma sordida* (Hemiptera: Reduviidae) from the Eastern and Western regions of Paraguay. Front Public Health.

[84] Monteiro F, Marcet P, Dorn P, Telleria J, Tibayrenc M (2010). Population genetics of triatomines. American trypanosomiasis Chagas disease, one hundred years of research.

[85] Shahzad A, Tian FY, John L, Joseph CS (2018). Effects of flexibility on the hovering performance of flapping wings with different shapes and aspect ratios. J Fluids Struct.

[86] Rajabi H, Gorb SN (2020). How do dragonfly wings work? A brief guide to functional roles of wing structural components. Int J Odonatol.

[87] Adámoli J, Torrella S, Herrera P, Castroviejo (2004). La expansión de la frontera agrícola y la conservación de la biodiversidad en el Chaco Argentino. Por la Biodiversidad en Latinoamérica.

[88] Dinerstein D, Olson D, Joshi A, Vynne A, Burgess ND, Wikramanayake E (2017). An ecoregion-based approach to protecting half the terrestrial realm. Bioscience.

