# Supplementary figures and images for: Relationship between genetic diversity and morpho-functional characteristics of flight-related traits in Triatoma garciabesi (Hemiptera: Reduviidae)
Source: Parasit Vectors. 2024 Mar 18;17:145. doi: 10.1186/s13071-024-06211-x (PMC10949591; doi:10.1186/s13071-024-06211-x)

# Kimura 2-p

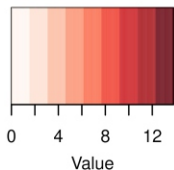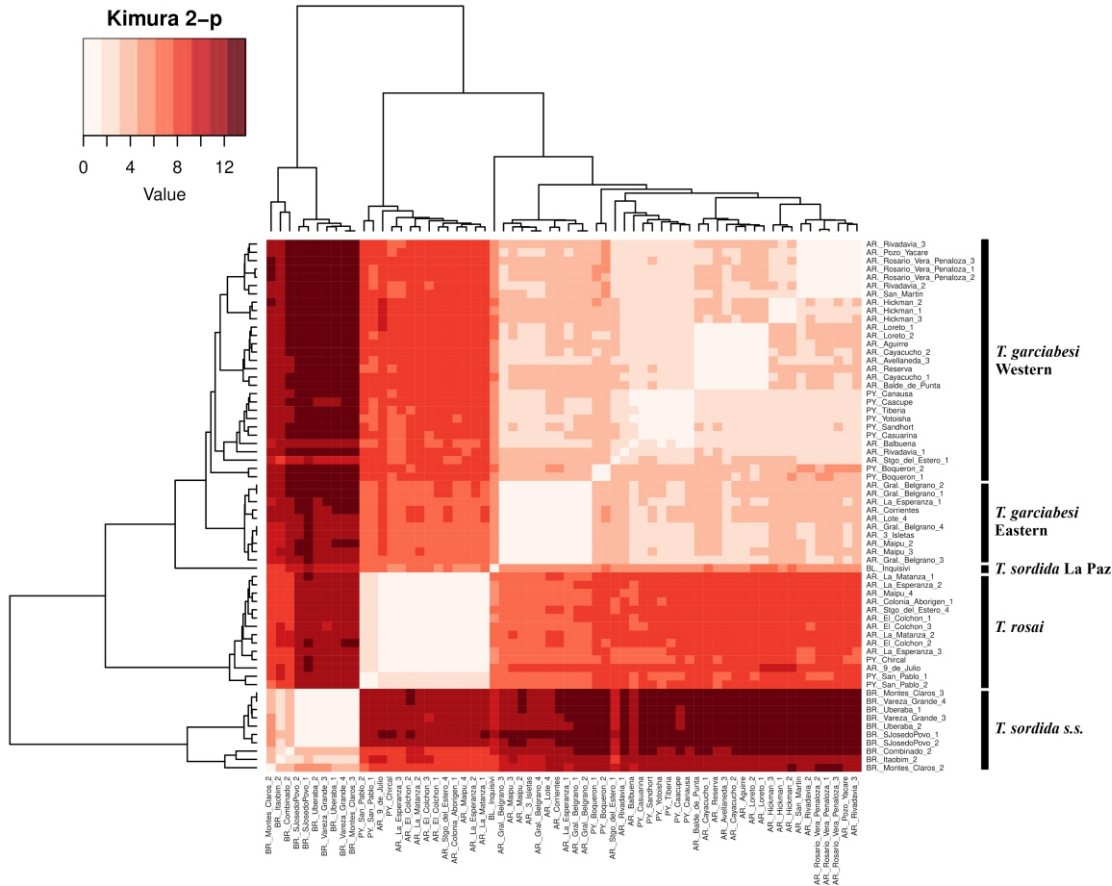

Supplement: Supplementary file 1 — Additional file 1: Figure S1. Heatmap representation of pairwise genetic distances obtained for coI gene fragments for T. garciabesi, T. rubroviaria, T. sordida s.s, T. rosai and T. sordida from La Paz, calculated under the k-2p substitution model. [file 13071_2024_6211_MOESM1_ESM.pdf]

# MDS T.sordida complex col

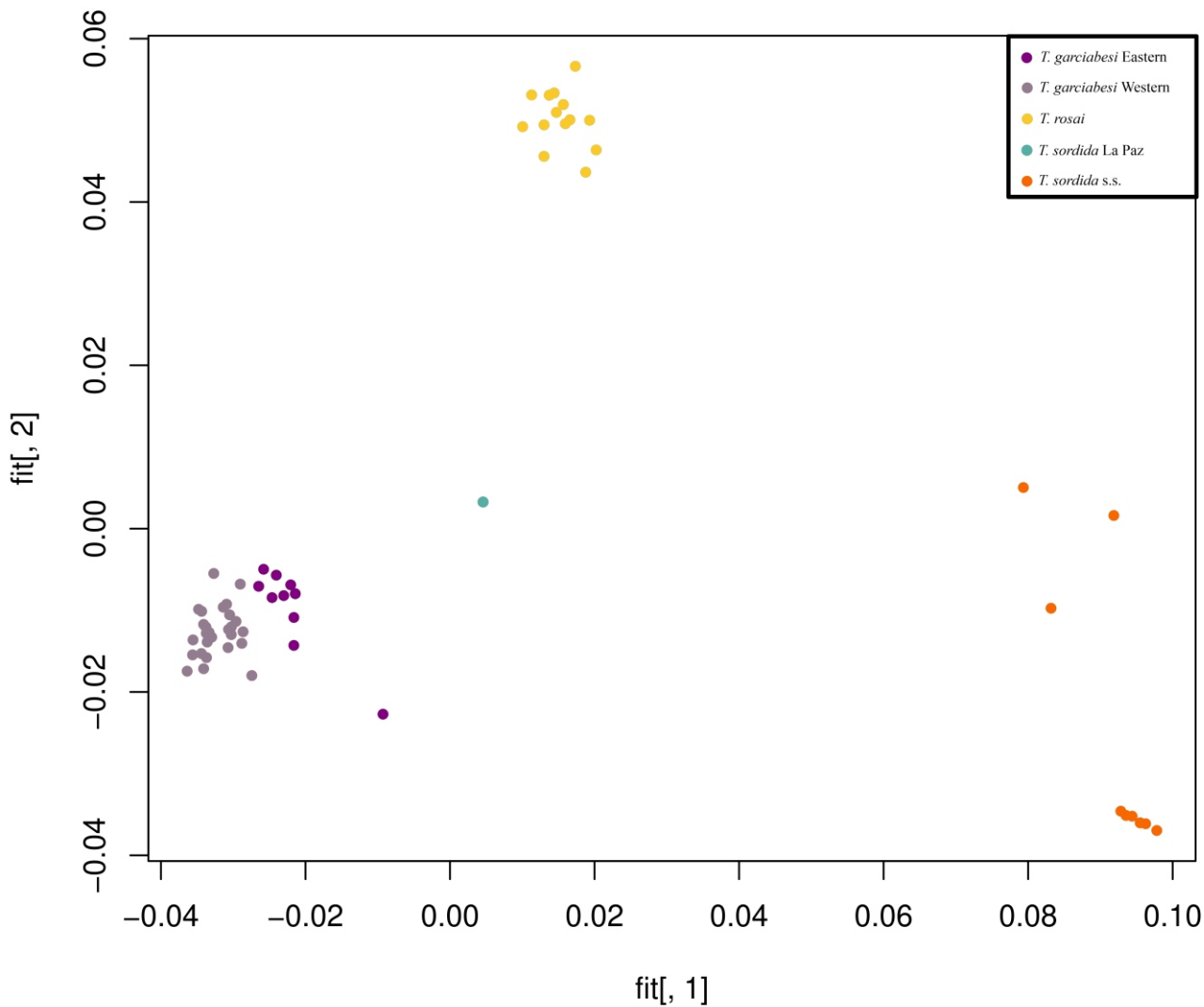

Supplement: Supplementary file 2 — Additional file 2: Figure S2. Graphical representation of the multidimensional scaling analysis (MDS) performed for genetic distance of coI gene fragments of T. garciabesi, T. rubroviaria, T. sordida s.s, T. rosai and T. sordida from La Paz. [file 13071_2024_6211_MOESM2_ESM.pdf]
