# Supplementary material for: Relationship between genetic diversity and morpho-functional characteristics of flight-related traits in Triatoma garciabesi (Hemiptera: Reduviidae)
Source: Parasit Vectors. 2024 Mar 18;17:145. doi: 10.1186/s13071-024-06211-x (PMC10949591; doi:10.1186/s13071-024-06211-x)
Supplement: Supplementary file 4 — Additional file 4: Table S2. Haplotype composition by frequency, lineage and individuals. [file 13071_2024_6211_MOESM4_ESM.pdf]

**Haplotype**	**Frequency**	**Lineage**	**Individuals**
H1	1	*T. sordida* La Paz	BL._Inquisivi
H2	1	*T. garciabesi* Western	PY._Boqueron_1
H3	1	*T. garciabesi* Western	PY._Boqueron_2
H4	1	*T. garciabesi* Eastern	AR._Gral._Belgrano_3
H5	2	*T. garciabesi* Eastern	AR._Gral._Belgrano_1, AR._Gral._Belgrano_2
H6	2	*T. garciabesi* Eastern	AR._3_Isletas, AR._Gral._Belgrano_4
H7	1	*T. garciabesi* Eastern	AR._Maipu_3
H8	1	*T. garciabesi* Eastern	AR._Corrientes
H9	1	*T. garciabesi* Eastern	AR._Lote_4
H10	1	*T. garciabesi* Eastern	AR._Maipu_2
H11	1	*T. garciabesi* Eastern	AR._La_Esperanza_1
H12	1	*T. garciabesi* Western	AR._Rivadavia_2
H13	1	*T. garciabesi* Western	AR._Rivadavia_3
H14	1	*T. garciabesi* Western	AR._San_Martin
H15	2	*T. garciabesi* Western	AR._Rosario_Vera_Penaloza_1, AR._Rosario_Vera_Penaloza_2
H16	1	*T. garciabesi* Western	AR._Rosario_Vera_Penaloza_3
H17	1	*T. garciabesi* Western	AR._Hickman_3
H18	1	*T. garciabesi* Western	AR._Hickman_1
H19	1	*T. garciabesi* Western	AR._Hickman_2
H20	1	*T. garciabesi* Western	AR._Reserva
H21	1	*T. garciabesi* Western	AR._Loreto_1
H22	1	*T. garciabesi* Western	AR._Avellaneda_3
H23	1	*T. garciabesi* Western	AR._Loreto_2
H24	1	*T. garciabesi* Western	AR._Aguirre
H25	1	*T. garciabesi* Western	AR._Rivadavia_1
H26	1	*T. garciabesi* Western	PY._Casuarina
H27	1	*T. garciabesi* Western	PY._Sandhort
H28	1	*T. garciabesi* Western	AR._Balbuena
H29	1	*T. garciabesi* Western	PY._Yotoisha
H30	1	*T. garciabesi* Western	PY._Caacupe
H31	1	*T. garciabesi* Western	PY._Tiberia
H32	1	*T. garciabesi* Western	PY._Canausa
H33	1	*T. garciabesi* Western	AR._Stgo_del_Estero_1
H34	1	*T. rosai*	PY._San_Pablo_1
H35	1	*T. rosai*	PY._San_Pablo_2
H36	1	*T. rosai*	AR._El_Colchon_3
H37	1	*T. rosai*	AR._La_Esperanza_2
H38	1	*T. rosai*	AR._Colonia_Aborigen_1
H39	1	*T. rosai*	PY._Chircal
H40	1	*T. rosai*	AR._La_Esperanza_3
H41	1	*T. rosai*	AR._La_Matanza_1
H42	1	*T. rosai*	AR._Maipu_4
H43	1	*T. rosai*	AR._Stgo_del_Estero_4

H44	1	*T. rosai*	AR._El_Colchon_1
H45	1	*T. rosai*	AR._El_Colchon_2
H46	1	*T. rosai*	AR._La_Matanza_2
H47	1	*T. rosai*	AR._9_de_Julio
H48	1	*T. sordida* s.s.	BR._Montes_Claros_2
H49	1	*T. sordida* s.s.	BR._SJosedoPovo_1
H50	2	*T. sordida* s.s.	BR._Montes_Claros_3, BR._Vareza_Grande_4
H51	1	*T. sordida* s.s.	BR._Vareza_Grande_3
H52	1	*T. sordida* s.s.	BR._SJosedoPovo_2
H53	1	*T. sordida* s.s.	BR._Uberaba_1
H54	1	*T. sordida* s.s.	BR._Uberaba_2
H55	1	*T. sordida* s.s.	BR._Combinado_2
H56	1	*T. sordida* s.s.	BR._Itaobim_2
H57	1	*T. garciabesi* Western	AR._Pozo_Yacare
H58	1	*T. garciabesi* Western	AR._Cayacucho_2
H59	1	*T. garciabesi* Western	AR._Cayacucho_1
H60	1	*T. garciabesi* Western	AR._Balde_de_Punta
